# Closed-loop insulin delivery for treatment of type 1 diabetes

**DOI:** 10.1186/1741-7015-9-120

**Published:** 2011-11-09

**Authors:** Daniela Elleri, David B Dunger, Roman Hovorka

**Affiliations:** 1 Department of Paediatrics and Institute of Metabolic Science, University of Cambridge, Hills Road, Cambridge, CB2 0QQ, UK

## Abstract

Type 1 diabetes is one of the most common endocrine problems in childhood and adolescence, and remains a serious chronic disorder with increased morbidity and mortality, and reduced quality of life. Technological innovations positively affect the management of type 1 diabetes. Closed-loop insulin delivery (artificial pancreas) is a recent medical innovation, aiming to reduce the risk of hypoglycemia while achieving tight control of glucose. Characterized by real-time glucose-responsive insulin administration, closed-loop systems combine glucose-sensing and insulin-delivery components. In the most viable and researched configuration, a disposable sensor measures interstitial glucose levels, which are fed into a control algorithm controlling delivery of a rapid-acting insulin analog into the subcutaneous tissue by an insulin pump. Research progress builds on an increasing use of insulin pumps and availability of glucose monitors. We review the current status of insulin delivery, focusing on clinical evaluations of closed-loop systems. Future goals are outlined, and benefits and limitations of closed-loop therapy contrasted. The clinical utility of these systems is constrained by inaccuracies in glucose sensing, inter- and intra-patient variability, and delays due to absorption of insulin from the subcutaneous tissue, all of which are being gradually addressed.

## Challenges for type 1 diabetes management

Type 1 diabetes is a chronic disease caused by T-cell-mediated autoimmune destruction of the pancreatic β cells in genetically predisposed individuals [1]. Insulin discovery in the early 1920s transformed diabetes from a uniformly fatal condition into a disease requiring life-long insulin-replacement therapy. The Diabetes Control and Complication Trial linked tight control of glucose to prevention of long-term diabetes-related vascular complications [2]. Intensification of insulin therapy has become an essential treatment method, but it has been hindered by an increased risk of hypoglycemia [3]. Severe hypoglycemia may lead to seizures or loss of consciousness, and can be life-threatening. Fear of hypoglycemia by patients and caregivers may adversely affect patients' quality of life and psychological well-being [4], and may result in 'over-compensatory' behaviors such as overeating or taking less insulin [5].

Technological innovations continue to benefit the management of type 1 diabetes. Novel rapid and long-acting insulin analogs and more physiological insulin-delivery systems including smart insulin pumps are increasingly being used. Glucose monitoring is evolving, leading to greater availability of subcutaneous continuous monitors, which provide frequent, real-time, and minimally invasive glucose measurements [6]. Sensor-augmented pump therapy increases convenience by integrating continuous glucose monitoring with an insulin pump. Despite these advances, the current best management of glucose control is still inadequate. Sensor-augmented pump therapy is unable to prevent severe hypoglycemia, with an incidence in patients using such therapy similar to that observed in those using conventional therapy [7]. Glucose levels in adults are above the target glucose level of 10 mmol/l for more than 8 hours per day, with even higher rates observed in the pediatric and adolescent populations [8]. Automated systems modulating insulin delivery according to sensor glucose levels, independently of patient supervision, may be needed to fully exploit existing glucose-sensing and insulin-delivery technologies, and in particular to protect against nocturnal hypoglycemia and minimize the impact of noncompliant behaviors.

In this review, we examine the current status of closed-loop insulin-delivery systems, extending previous work [9-12], while focusing on their clinical applications and evaluations. Future goals are outlined, and the benefits and limitations of closed-loop therapy contrasted.

## Closed-loop insulin delivery

### The artificial pancreas

**Figure 1:**
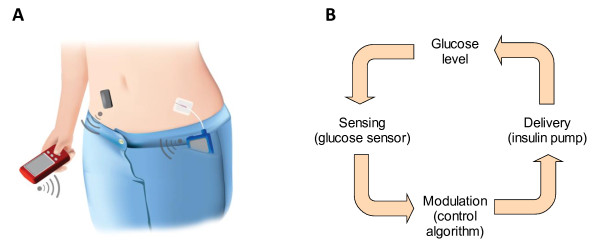
**An illustrative representation of a closed-loop insulin delivery system**. **(A) **A sensor (black rectangle) transmits information about interstitial glucose levels to a handheld device about the size of a cellphone (red box) which holds a control algorithm and interacts with the user. An insulin pump (blue box in the pocket) delivers a rapid-acting insulin analog subcutaneously. Insulin delivery is modulated by the control algorithm. The communication between the system components is wireless. The control algorithm can also reside within the insulin pump. (Adapted from Hovorka [12]). **(B) **The closed loop replicates the physiological feedback normally provided by the β-cell.

Closed-loop insulin delivery, also referred to as the artificial pancreas, is an emerging therapeutic approach for people with type 1 diabetes. It is a medical device consisting of a linked continuous glucose monitor and an insulin pump. Wireless communication facilitates automated data transfer between components without the need for human intervention. A schematic view of the artificial pancreas and the physiological feedback normally provided by the β-cell is shown in Figure 1.

The novelty of this approach resides in the real-time feedback between glucose levels and insulin delivery, similar to that presented by the β-cell. Insulin delivery is modulated at intervals of 1 to 15 minutes, depending on interstitial glucose levels, in contrast to the pre-programmed insulin delivery that takes place during conventional insulin pump treatment.

The key component of the artificial pancreas is the control algorithm, which directs insulin delivery according to glucose levels while accounting for inherent measurement errors and kinetic delays. Various algorithms have been developed [13], but two main categories are the most relevant: the proportional-integral-derivative control (PID) [14,15] and the model-predictive control (MPC) [16-18].

PID algorithms adjust insulin delivery by considering deviations from a target glucose level (proportional component), the area under the curve between the measured and the target glucose level (integral component), and the rate of change in the measured glucose levels (derivative component) [15].

MPC algorithms, by contrast, employ a mathematical model of human glucose regulation to link insulin delivery and glucose excursions as described in numerous theoretical, animal, and computer-simulation studies [16,18-22]. Insulin delivery is calculated by minimizing the difference between forecasted glucose concentrations and the target glucose levels over a prediction window of 1.5 to 3 hours, or longer.

MPC algorithms can be regarded as proactive; they forecast glucose levels in anticipation of the glucose-lowering effect of administered insulin and of announced disturbances such as meals and physical activity. PID algorithms, by contrast, can be considered reactive, as they respond to observed glucose levels and are less equipped to handle announced meals and patient-directed insulin boluses. The safety of control algorithms can be enhanced by a supervisory module, which constrains insulin delivery by limiting the maximum insulin rate or by suspending insulin delivery when glucose levels are low or decreasing rapidly [23,24].

The MPC method is well suited to compensate for time delays associated with the subcutaneous route of insulin administration and interstitial glucose measurements. Other algorithms have been tested clinically, such as fuzzy logic, which is developed from qualitative approximations of clinical judgment by diabetes practitioners [25], a combination of MPC and PID algorithms for insulin and glucagon delivery [19], or a PID with insulin feedback [26]. One of the objectives of the current research is to integrate existing control algorithms within increasingly sophisticated insulin pumps and continuous glucose monitors.

### Progress thus far

It is anticipated that the artificial pancreas will evolve with increasing technology sophistication and more comprehensive treatment objectives [27] (Table 1). Early generations of the artificial pancreas are likely to provide benefits in terms of reduced incidence of hypoglycemia. Benefits may be population-specific; for example, compliant, motivated subjects may benefit from a reduced risk of hypoglycemia whereas less compliant subjects, including adolescents, may benefit from reduced glucose levels. Follow-up closed-loop applications may address hyperglycemia, postprandial control and other lifestyle changes, including exercise. Meals and exercise can be 'announced' to the control algorithm, and prandial insulin boluses can be delivered in the conventional way simplifying closed-loop operation. In a more challenging 'fully closed-loop' configuration, the control algorithm is not aware of meals and exercise, and delivers insulin solely based on sensor glucose levels. Glucagon coadministration can be used to counteract peripheral overinsulization following insulin boluses or delayed insulin absorption.

**Table 1:** Closed-loop approaches according to treatment objective

Objective	Insulin-delivery modulation
Reduce severity and/or duration of hypoglycemia	Suspension of insulin delivery at hypoglycemia (low glucose suspend)
Hypoglycemia prevention	Pre-emptive suspension/reduction of insulin delivery before hypoglycemia occurs
Control to range	Modulation (increase or decrease) of insulin delivery outside target range to limit hypoglycemia and hyperglycemic excursions
Overnight glucose control	Modulation of insulin delivery for nocturnal glucose control; lifestyle disturbances have limited effect
Closed-loop system with meal/exercise announcement	Modulation of insulin delivery after meals using boluses administered by patient with announcement of these, and exercise to the algorithm
Fully closed-loop system	Modulation of insulin delivery when the control algorithm is unaware of meals, exercise, stress and other lifestyle disturbances that affect glucose control; glucagon may be coadministered to reduce risk of hypoglycemia

Apart from the low glucose suspend (LGS) approach described below, which has entered postmarketing stage, all other approaches are under investigation in controlled laboratory conditions with realistic plans to perform studies under free-living conditions. Table 2 outlines the status and achievements of various closed-loop approaches.

**Table 2:** Summary of achieved results

Objective/approach	Status	Results	**Ref**.
Low glucose suspend	Postmarketing studies	Reduced nocturnal hypoglycemia in those with greatest risk; well- accepted by patients	[61,62]
Suspend to prevent low glucose	Laboratory studies; home studies planned	Prevention of 80% of events of nocturnal hypoglycemia; effective as part of overnight closed-loop system	[39,40]
Treat to range	Laboratory testing underway	-	-
Overnight	Laboratory studies; home studies planned	Increased time spent in target glucose range by 20% in adolescents and adults; reduced risk of nocturnal hypoglycemia	[24,43]
Meal announcement	Laboratory studies	Feasibility documented in children, adults, and pregnant women using various control algorithms; preferred option by most investigators	[26,44,46,58,63]
Fully closed-loop	Laboratory studies	Feasibility documented in children and adults; addition of small prandial bolus improves control; delayed insulin absorption/action remains a challenge	[25,45,64]
Fully closed-loop with glucagon coadministration	Laboratory studies	Feasibility documented in adults; glucagon helpful but cannot always overcome insulin overdelivery	[47,48]

### Approaches to reduce incidence of hypoglycemia

#### Low glucose suspend

Hypoglycemia associated with low sensor-measured glucose levels sustained for 2 to 4 hours may lead to seizures [28]. The body's defensive mechanisms against hypoglycemia are impaired during the night in people with type 1 diabetes, who have lost the ability to release the appropriate counter-regulatory hormones [29-32]. The simplest approach to reduce severity of hypoglycemia is to interrupt insulin delivery. The LGS function was the first example of a commercial application of closed-loop insulin delivery. An insulin pump with an integrated continuous glucose monitoring (CGM) (Paradigm^® ^Veo; Medtronic Diabetes, Northridge, CA, USA) automatically suspends insulin delivery for up to 2 hours when hypoglycemia is detected and the hypoglycemia alarm is not acknowledged by the patient [33]. Patients may be unconscious during hypoglycemia, and their ability to respond to alarms is reduced. Thus, a considerable safety benefit may be obtained from the LGS function. However, concerns have been raised about the attendant hyperglycemia that can result, especially from false-positive hypoglycemia detection. The hyperglycemia risk is not negligible, but thus far only mild rebound hyperglycemia and minimal ketonaemia have been reported after a temporary suspension of insulin administration [34-38].

The LGS function aims to reduce the severity of hypoglycemia, but does not prevent it, which was the objective of work by Buckingham *et al*., who developed and demonstrated, in laboratory settings, the effectiveness of an algorithm to discontinue insulin delivery when pending hypoglycemia was predicted [39]. This approach was investigated in adults in a clinical setting. Using a pump shut-off time of 90 minutes and a glucose threshold of 4.4 mmol/l, 56% of hypoglycemic episodes were prevented at a prediction horizon of 30 minutes, and 80% when the horizon was extended to 45 minutes. This approach was then tested overnight in young people, in whom hypoglycemia was induced by gradually increasing the subcutaneous insulin delivery [40]. Hypoglycemia was prevented in up to 84% of cases, using five prediction algorithms. All these prediction algorithms used a 35-minute prediction horizon to allow time for the pump suspension to be effective in lowering insulin levels once basal infusion was suspended.

#### Overnight closed-loop

Most severe hypoglycemic episodes occur during sleep between midnight and 8 am [41]. As overnight glucose control is not complicated by meals or physical activity, closed-loop could help prevent nocturnal hypoglycemia. This is a common clinical problem of great concern to parents and carers of children with type 1 diabetes [42].

Over the past 4 years, diabetes research at Cambridge University has focused on the development and testing of overnight closed-loop insulin delivery systems. Clinical studies have been performed in children, adults and pregnant women [24,43,44], evaluating various scenarios to reproduce real-life challenges for overnight glucose control, which could potentially predispose to nocturnal hypoglycemia, such as afternoon exercise or the consumption of alcohol. Evening meals of different sizes and compositions were also tested. An MPC algorithm was used to determine basal insulin delivery according to sensor glucose readings, whereas prandial insulin boluses were administered based on the subjects' standard practice. Most of those clinical studies adopted a randomized crossover design comparing closed-loop insulin delivery with the conventional insulin pump therapy.

**Figure 2:**
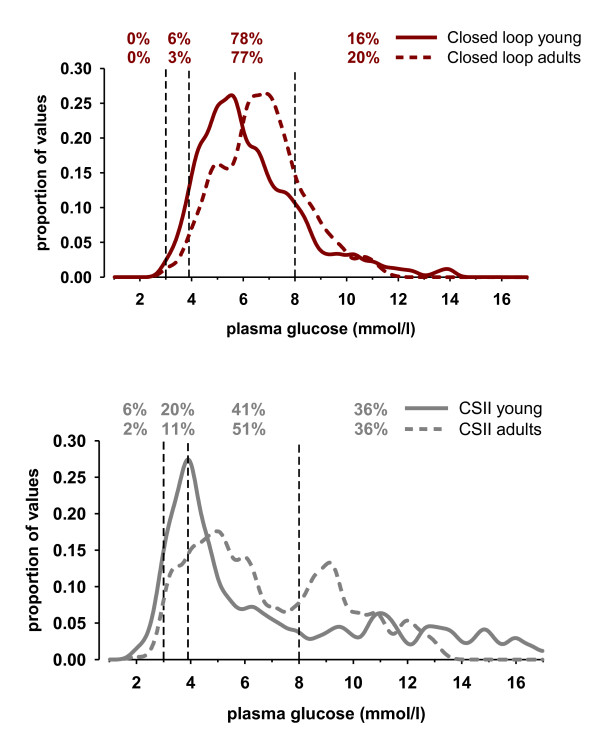
**Distribution of plasma glucose levels after midnight in young people and adults during (top panel) closed-loop and (bottom panel) conventional insulin-pump therapy (continuous subcutaneous insulin infusion (CSII))**. Vertical dashed lines indicate the threshold of significant hypoglycemia (3.0 mmol/l) and the target glucose range of 3.91 to 8.0 mmol/l. Values at the top denote the percentage of plasma glucose values within the respective glucose ranges (reproduced with permission from Kumareswaran *et al*. [60]).

In these randomized crossover studies, the overnight closed-loop system significantly increased the percentage of time that plasma glucose levels were within a target range of 3.9 to 8.0 mmol/l in children and adolescents (from 40% to 60%, *P *= 0.002) and in adults (from 50% to 76%, *P *< 0.001). The effectiveness of the closed-loop system was most pronounced after midnight, when the system became fully effective. Combined results of both young and adult patients after midnight during closed-loop and the conventional pump therapy are summarized in Figure 2. Notably, these results indicate that the closed-loop system resulted in a significantly reduced time spent with glucose below the target range of 3.9 mmol/l both in young (from 4.1% to 2.1%, *P *= 0.03) and adult (from 6.7% to 2.8%, *P *= 0.04) patients.

#### Fully closed-loop versus closed-loop with meal announcement

Two main closed-loop approaches have been adopted in the clinical studies for prandial insulin delivery: 'fully closed-loop' and 'closed-loop with meal announcement' [9]. A fully closed-loop system delivers insulin without information about the size or time of meals, whereas information about meals together with information about manually administered prandial insulin boluses is provided to closed-loop systems adopting the 'meal announcement' approach. In a hybrid system, the delivery of pre-meal insulin boluses remains one of the tasks for which patients are responsible, with the closed-loop system automatically determining the insulin delivery between meals [27].

The hybrid system with meal-time priming boluses has been shown to reduce postprandial hyperglycemia compared to a fully closed-loop system [45]. As delays in insulin absorption of the order of 30 to 100 minutes are a major challenge for safe and effective postprandial glucose control, this hybrid approach may be considered a transition step from 'closed-loop with meal announcement'.

The feasibility and efficacy of MPC-based closed-loop insulin delivery was also recently demonstrated in women with type 1 diabetes throughout different stages of pregnancy [44]. Near-optimal nocturnal glycemic control was obtained with the closed-loop system both in early and late pregnancy, coping well with both the longitudinal changes in insulin requirements and the insulin sensitivity associated with pregnancy. Studies of a well-controlled cohort of pregnant women suggested a reduced risk of very low glucose levels with closed-loop insulin delivery, but otherwise similar glucose control [46].

Glucagon coadministration has also been investigated with the fully closed-loop approach [47,48]. Although effective in reducing the risk of hypoglycemia, glucagon cannot be fully relied upon to reverse the effect of insulin overdelivery.

### Towards home studies

#### *In silico *testing

Evaluation of closed-loop systems in a controlled laboratory setting is an essential step to assess safety and efficacy before moving to home settings, where everyday life poses additional challenges for effective closed-loop performance. *In silico *studies play a crucial role in complementing clinical testing, and address scenarios that cannot, for practical or ethical reasons, be tested in clinical studies [49-51].

A computer-based simulation environment developed at Cambridge University builds on a model of glucose regulation, glucose sensing and insulin-pump delivery. It includes a virtual population of subjects with type 1 diabetes with a range of insulin-sensitivity and glucoregulatory parameters, to represent both inter-subject and intra-subject variability [49] Pre-clinical *in silico *testing enables us to assess scenarios that are expected to occur rarely but could be potentially harmful. For example, simulations can evaluate the system's response to sensor-data dropouts, large calibration errors, communication failures, unannounced meals, or errors in carbohydrate estimation and thus bolus dosing [51]. Different CGM devices or different algorithms can be tested. Another simulator has been accepted by the US Food and Drug Administration to replace animal testing, and has been widely used for this purpose [50]. The simulator developed by the Medtronic team [52] has complemented animal testing, and has been instrumental in tuning the Medtronic PID algorithm [26,45].

#### Transitional and early home studies

Transition to the outpatient studies could be performed gradually with an intermediate phase under supervision of the research team in the subject's home, or at a transitional clinical or hotel-like research facility. The transition phase needs to emphasize education to operate the closed-loop system correctly and confidently. Competency-assessment tools may be used to ensure subjects' knowledge and ability. User acceptance and user friendliness are important aspects that may affect study results, quality of life, and ultimately technology adoption. Subjects' satisfaction may be assessed by the use of questionnaires or qualitative interviews [53].

Home testing of overnight closed-loop is feasible with present technologies. Pilot studies may evaluate efficacy, safety and utility over a short period of time, whereas longer studies are needed to demonstrate effectiveness on glycemic control and rates of hypoglycemia. Specific challenges of day-time glucose control, such as meal intake and exercise, will need to be considered when moving to a full-time (24 hours a day, 7 days a week) closed-loop system.

### Expectations for clinical practice

The main goal of closed-loop therapy is to achieve good glycemic control while reducing the risk of hypoglycemia in people with type 1 diabetes. Although reduction in HbA1c levels is a common key outcome expected by the regulatory authorities, it is important to consider that for some patients, avoidance of severe recurrent hypoglycemia may be the more important focus. Improvement in glycemic variability could be an important target for those subjects with acceptable HbA1c and low incidence of severe hypoglycemia. Results thus far indicate that these targets may be achieved with closed-loop therapy.

Despite the perceived benefits of closed-loop therapy, it is essential to set realistic goals to keep up with reasonable clinical expectations. Furthermore, the impact of this novel treatment tool on quality of life will need to be assessed. Current closed-loop systems are limited by suboptimal performance of the available system components, which include CGM devices, insulin formulations, and insulin-delivery systems. For example, the accuracy of commercially available CGM devices is impaired by errors arising from incorrect calibration, rapid glucose changes, and glucose levels within the hypoglycemic range. Sensor failures may lead to termination of closed-loop operation. Similarly, reduced accuracy of insulin delivery at low volumes by current insulin pumps could negatively affect the performance of closed-loop systems in patients with low insulin requirements. The reliability of wireless communication between the components also needs to be addressed.

Patient engagement in the daily use of closed-loop systems is also of importance. It is possible that the introduction of the artificial pancreas as an integral part of diabetes management will change current clinical practice. Although patients could be released from the constant attention to insulin adjustments they presently experience, their active engagement will still be needed during prolonged exercise, illness, puberty or pregnancy, which are associated with significant changes in insulin sensitivity and requirements. For this reason, specific training and education in the use of closed-loop therapy will be needed by both patients and healthcare professionals. The benefits and limitations of the system will also need to be clarified.

### The future: perfecting closed-loop therapy

For closed-loop insulin delivery to be used widely, improvements may need to increase effectiveness, safety, and convenience gradually (Table 3). Specific challenges include postprandial glucose control. Improved understanding of postprandial glucose fluctuations will provide additional information to refine control algorithms. Research using stable glucose isotopes to measure gut absorption of meals of different sizes or compositions is underway [54]. A dual-hormone closed-loop approach is also under investigation using, for example, pramlintide, an amylin analog that is normally released by β-cells along with insulin after a meal. Amylin delays glucose absorption, thus improving post-meal glucose control [55]. Similarly, a more physiologic approach will be needed to cope with exercise-related changes in insulin sensitivity.

**Table 3:** Goals to improve gradually closed-loop performance

Factor	Desirable improvements
Insulin delivery	Faster absorption
Glucose sensing	Increased accuracy and reliability; reduced false-positive hypoglycemia alarms
Insulin modulation	Adaptive algorithms
Dual-hormone systems	Dual-chamber pumps
Communication between glucose sensor and insulin pump	More reliable connectivity; standardized communication protocol
Human factors	Reduced size of devices; enhanced ease of use; sensing and delivery from a single body port

A major turning point in the development of closed-loop systems will be the introduction of insulin formulations with faster absorption. With rapid-acting insulin analogs, the maximum blood-glucose-lowering effect occurs after up to 90 to 120 min. This lag constitutes one of the greatest challenges for closed-loop systems, and availability of faster insulin analogs or devices accelerating insulin absorption will eventually translate into more effective and safer insulin delivery. These pharmaceutical developments are ongoing [56,57].

Engineering factors needing further attention include accurate insulin delivery at low rates, more reliable CGM devices, dependable wireless communication, and improved control algorithms. Human factors should consider usability, safety and training/education components. The availability of smaller and more user-friendly devices might be particularly important for children [42]. Telemonitoring is also seen as an additional feature to enhance safety, although its practicability and utility are yet to be established. The clinical team can be informed about system malfunctions, subject compliance, and the level of glucose control, with the possibility of remote algorithm updates.

An alternative to subcutaneous insulin delivery is intraperitoneal insulin infusion, which restores the portal/peripheral insulin concentration gradient. Renard *et al*. evaluated the feasibility and efficacy of a closed-loop system with an implantable insulin pump coupled to a subcutaneous glucose sensor [58]. Further progress could be made with an intraperitoneal port to bypass the limitations of implantable insulin pumps [59].

## Conclusions

Closed-loop insulin delivery presents a tangible treatment option and may serve as a bridge to a cure for type 1 diabetes until stem-cell therapy or similar long-term biologic interventions become available. Closed-loop insulin delivery may revolutionize not only the way diabetes is managed but also patients' perceptions of living with diabetes, by reducing the burden on patients and carers, and their fears of complications related to diabetes, including those associated with low and high glucose levels. The next step is to confirm the encouraging results collected under controlled laboratory settings in real-life conditions at home.

## Competing interests

RH has received speaker honoraria from Minimed Medtronic, Lifescan, Eli Lilly, and Novo Nordisk, has served on an advisory panel for Animas and Minimed Medtronic, has received license fees from BBraun and Beckton Dickinson; and has served as a consultant to Beckton Dickinson, BBraun and Profil. DE has no competing financial interests. RH and DBD have patent applications pending.

## Authors' contributions

DE researched data and drafted the report. RH and DBD contributed to the interpretation of the data, and the writing and critical review of the report. All authors read and approved the final manuscript.

## Authors' information

Daniela Elleri, MD, is a clinical research fellow in Paediatrics at the Institute of Metabolic Science and Department of Paediatrics, University of Cambridge, UK, who is actively involved in the clinical research studies evaluating closed-loop insulin-delivery systems in people with type 1 diabetes. David B Dunger, MD, FRCP and FRCPC, is a Professor of Paediatrics at Addenbrooke's Hospital, University of Cambridge, with has a particular interest in the pathophysiology of diabetes during childhood and adolescence, and the genetic and environmental interactions that determine size at birth and childhood growth. His research group are coordinating the follow-up of large pediatric cohorts designed to investigate the genetics and pathophysiology of diabetes and its complications. Roman Hovorka, BSc, MSc, and PhD, is Principal Research Associate at the Institute of Metabolic Science and Department of Paediatrics, University of Cambridge, UK. His current research interests include evaluation of medical technologies to support diagnosis and treatment of diabetes and related metabolic diseases. He leads research team developing and testing closed-loop insulin delivery systems in type 1 diabetes, and he also works on developing approaches for glycemic control in the critically ill.

## Pre-publication history

The pre-publication history for this paper can be accessed here:

http://www.biomedcentral.com/1741-7015/9/120/prepub

## References

[B1] Todd JA Etiology of type 1 diabetes Immunity 2010 32 457 467 10.1016/j.immuni.2010.04.001 20412756

[B2] Diabetes Control and Complication Trial Study Group (DCCT) The effect of intensive treatment of diabetes on the development and progression of long-term complications in insulin-dependent diabetes-mellitus N Engl J Med 1993 329 977 986 8366922 10.1056/NEJM199309303291401

[B3] Cryer PE The barrier of hypoglycemia in diabetes Diabetes 2008 57 3169 3176 10.2337/db08-1084 19033403 PMC2584119

[B4] Nixon R Pickup JC Fear of hypoglycemia in type 1 diabetes managed by continuous subcutaneous insulin infusion: is it associated with poor glycemic control? Diabetes Technol Ther 2011 13 93 98 10.1089/dia.2010.0192 21284474

[B5] Wild D Von Maltzahn R Brohan E Christensen T Clauson P Gonder-Frederick L A critical review of the literature on fear of hypoglycemia in diabetes: Implications for diabetes management and patient education Patient Educ Couns 2007 68 10 15 10.1016/j.pec.2007.05.003 17582726

[B6] Klonoff DC Continuous glucose monitoring: roadmap for 21st century diabetes therapy Diabetes Care 2005 28 1231 1239 10.2337/diacare.28.5.1231 15855600

[B7] Bergenstal RM Tamborlane WV Ahmann A Buse JB Dailey G Davis SN Joyce C Peoples T Perkins BA Welsh JB Willi SM Wood MA Effectiveness of sensor-augmented insulin-pump therapy in type 1 diabetes N Engl J Med 2010 363 311 320 10.1056/NEJMoa1002853 20587585

[B8] Tamborlane WV Beck RW Bode BW Buckingham B Chase HP Clemons R Fiallo-Scharer R Fox LA Gilliam LK Hirsch IB Huang ES Kollman C Kowalski AJ Laffel L Lawrence JM Lee J Mauras N O'Grady M Ruedy KJ Tansey M Tsalikian E Weinzimer S Wilson DM Wolpert H Wysocki T Xing D Continuous glucose monitoring and intensive treatment of type 1 diabetes N Engl J Med 2008 359 1464 1476 18779236 10.1056/NEJMoa0805017

[B9] Hovorka R Continuous glucose monitoring and closed-loop systems Diabet Med 2006 23 1 12 16409558 10.1111/j.1464-5491.2005.01672.x

[B10] Steil GM Rebrin K Closed-loop insulin delivery - what lies between where we are and where we are going? Expert Opin Drug Deliv 2005 2 353 362 10.1517/17425247.2.2.353 16296759

[B11] Renard E Costalat G Chevassus H Bringer J Artificial beta-cell: clinical experience toward an implantable closed-loop insulin delivery system Diabetes Metab 2006 32 497 502 10.1016/S1262-3636(06)72802-6 17130808

[B12] Hovorka R Closed-loop insulin delivery: from bench to clinical practice Nat Rev Endocrinol 2011 7 385 395 10.1038/nrendo.2011.32 21343892

[B13] Bequette BW A critical assessment of algorithms and challenges in the development of a closed-loop artificial pancreas Diabetes Technol Ther 2005 7 28 47 10.1089/dia.2005.7.28 15738702

[B14] Marchetti G Barolo M Jovanovic L Zisser H Seborg DE An improved PID switching control strategy for type 1 diabetes IEEE Trans Biomed Eng 2008 55 857 865 18334377 10.1109/TBME.2008.915665

[B15] Steil GM Panteleon AE Rebrin K Closed-loop insulin delivery-the path to physiological glucose control Adv Drug Deliv Rev 2004 56 125 144 10.1016/j.addr.2003.08.011 14741112

[B16] Hovorka R Canonico V Chassin LJ Haueter U Massi-Benedetti M Orsini FM Pieber TR Schaller HC Schaupp L Vering T Wilinska ME Nonlinear model predictive control of glucose concentration in subjects with type 1 diabetes Physiol Meas 2004 25 905 920 10.1088/0967-3334/25/4/010 15382830

[B17] Magni L Raimondo DM Bossi L Man CD De NG Kovatchev B Cobelli C Model predictive control of type 1 diabetes: an in silico trial J Diabetes Sci Technol 2007 1 804 812 19885152 10.1177/193229680700100603 PMC2769684

[B18] Parker RS Doyle FJ III Peppas NA A model-based algorithm for blood glucose control in type I diabetic patients IEEE Trans Biomed Eng 1999 46 148 157 10.1109/10.740877 9932336

[B19] El-Khatib FH Jiang J Damiano ER Adaptive closed-loop control provides blood-glucose regulation using dual subcutaneous insulin and glucagon infusion in diabetic swine J Diabetes Sci Technol 2007 1 181 192 19888405 10.1177/193229680700100208 PMC2771467

[B20] Lee H Buckingham BA Wilson DM Bequette BW A closed-loop artificial pancreas using model predictive control and a sliding meal size estimator J Diabetes Sci Technol 2009 3 1082 1090 20144421 10.1177/193229680900300511 PMC2769914

[B21] Wang Y Dassau E Doyle FJ III Closed-loop control of artificial pancreatic Beta -cell in type 1 diabetes mellitus using model predictive iterative learning control IEEE Trans Biomed Eng 2010 57 211 219 19527957 10.1109/TBME.2009.2024409

[B22] Grosman B Dassau E Zisser HC Jovanovic L Doyle FJ III Zone model predictive control: a strategy to minimize hyper- and hypoglycemic events J Diabetes Sci Technol 2010 4 961 975 20663463 10.1177/193229681000400428 PMC2909531

[B23] Kovatchev B Patek S Dassau E Doyle FJ III Magni L De NG Cobelli C Control to range for diabetes: functionality and modular architecture J Diabetes Sci Technol 2009 3 1058 1065 20144419 10.1177/193229680900300509 PMC2769910

[B24] Hovorka R Allen JM Elleri D Chassin LJ Harris J Xing D Kollman C Hovorka T Larsen AM Nodale M De PA Wilinska ME Acerini CL Dunger DB Manual closed-loop insulin delivery in children and adolescents with type 1 diabetes: a phase 2 randomised crossover trial Lancet 2010 375 743 751 10.1016/S0140-6736(09)61998-X 20138357

[B25] Atlas E Nimri R Miller S Grunberg EA Phillip M MD-logic artificial pancreas system: a pilot study in adults with type 1 diabetes Diabetes Care 2010 33 1072 1076 10.2337/dc09-1830 20150292 PMC2858178

[B26] Steil GM Palerm CC Kurtz N Voskanyan G Roy A Paz S Kandeel FR The effect of insulin feedback on closed loop glucose control J Clin Endocrinol Metab 2011 96 1402 1408 10.1210/jc.2010-2578 21367930 PMC3085208

[B27] Kowalski AJ Can we really close the loop and how soon? Accelerating the availability of an artificial pancreas: a roadmap to better diabetes outcomes Diabetes Technol Ther 2009 11 S113 S119 10.1089/dia.2008.0021 19621478

[B28] Buckingham B Wilson DM Lecher T Hanas R Kaiserman K Cameron F Duration of nocturnal hypoglycemia before seizures Diabetes Care 2008 31 2110 2112 10.2337/dc08-0863 18694975 PMC2571056

[B29] Cryer PE Glucose counterregulation in normal and diabetic man Clin Physiol 1985 5 Suppl 5 61 65 3914389

[B30] Cryer PE Mechanisms of hypoglycemia-associated autonomic failure and its component syndromes in diabetes Diabetes 2005 54 3592 3601 10.2337/diabetes.54.12.3592 16306382

[B31] Simonson DC Tamborlane WV DeFronzo RA Sherwin RS Intensive insulin therapy reduces counterregulatory hormone responses to hypoglycemia in patients with type I diabetes. Ann Intern Med 1985 103 184 190 10.7326/0003-4819-103-2-184 3893253

[B32] Jones TW Porter P Sherwin RS Davis EA O'Leary P Frazer F Byrne G Stick S Tamborlane WV Decreased epinephrine responses to hypoglycemia during sleep N Engl J Med 1998 338 1657 1662 10.1056/NEJM199806043382303 9614256

[B33] Pickup JC Semi-closed-loop insulin delivery systems: early experience with low-glucose insulin suspend pumps Diabetes Technol Ther 2011 13 695 698 10.1089/dia.2011.0103 21668344

[B34] Attia N Jones TW Holcombe J Tamborlane WV Comparison of human regular and lispro insulins after interruption of continuous subcutaneous insulin infusion and in the treatment of acutely decompensated IDDM Diabetes Care 1998 21 817 821 10.2337/diacare.21.5.817 9589247

[B35] Guerci B Meyer L Salle A Charrie A Dousset B Ziegler O Drouin P Comparison of metabolic deterioration between insulin analog and regular insulin after a 5-hour interruption of a continuous subcutaneous insulin infusion in type 1 diabetic patients J Clin Endocrinol Metab 1999 84 2673 2678 10.1210/jc.84.8.2673 10443658

[B36] Krzentowski G Scheen A Castillo M Luyckx AS Lefebvre PJ A 6-hour nocturnal interruption of a continuous subcutaneous insulin infusion. Metabolic and hormonal consequences and scheme for a prompt return to adequate control Diabetologia 1983 24 314 318 6347779 10.1007/BF00251815

[B37] Pickup JC Viberti GC Bilous RW Keen H Alberti KGMM Home PD Binder C Safety of continuous subcutaneous insulin infusion. Metabolic deterioration and glycemic auto-regulation after deliberate cessation of infusion Diabetologia 1982 22 175 179 7042428 10.1007/BF00283748

[B38] Scheen A Castillo M Jandrain B Krzentowski G Henrivaux P Luyckx A Lefebvre P A 2-hour nocturnal interruption of continuous subcutaneous insulin infusion induces a delayed and sustained metabolic deterioration in C-peptide negative type-1 (insulin-dependent) diabetic patients Diabetologia 1983 25 192 192

[B39] Buckingham B Cobry E Clinton P Gage V Caswell K Kunselman E Cameron F Chase HP Preventing hypoglycemia using predictive alarm algorithms and insulin pump suspension Diabetes Technol Ther 2009 11 93 97 10.1089/dia.2008.0032 19848575 PMC2979338

[B40] Buckingham B Chase HP Dassau E Cobry E Clinton P Gage V Caswell K Wilkinson J Cameron F Lee H Bequette BW Doyle FJ III Prevention of nocturnal hypoglycemia using predictive alarm algorithms and insulin pump suspension Diabetes Care 2010 33 1013 1017 10.2337/dc09-2303 20200307 PMC2858164

[B41] Diabetes Control and Complication Trial Study Group (DCCT) Epidemiology of severe hypoglycemia in the diabetes control and complications trial Am J Med 1991 90 450 459 2012085

[B42] Elleri D Acerini CL Allen JM Hayes J Pesterfield C Wilinska ME Dunger DB Hovorka R Parental attitudes towards overnight closed-loop glucose control in children with type 1 diabetes Diabetes Technol Ther 2010 12 35 39 10.1089/dia.2009.0084 20082583

[B43] Hovorka R Kumareswaran K Harris J Allen JM Elleri D Xing D Kollman C Nodale M Murphy HR Dunger DB Amiel SA Heller SR Wilinska ME Evans ML Overnight closed loop insulin delivery (artificial pancreas) in adults with type 1 diabetes: crossover randomised controlled studies BMJ 2011 342 d1855 10.1136/bmj.d1855 21493665 PMC3077739

[B44] Murphy HR Elleri D Allen JM Harris J Simmons D Rayman G Temple R Dunger DB Haidar A Nodale M Wilinska ME Hovorka R Closed-loop insulin delivery during pregnancy complicated by type 1 diabetes Diabetes Care 2011 34 406 411 10.2337/dc10-1796 21216859 PMC3024358

[B45] Weinzimer SA Steil GM Swan KL Dziura J Kurtz N Tamborlane WV Fully automated closed-loop insulin delivery versus semi-automated hybrid control in pediatric patients with type 1 diabetes using an artificial pancreas Diabetes Care 2008 31 934 939 10.2337/dc07-1967 18252903

[B46] Murphy HR Kumareswaran K Elleri D Allen JM Caldwell K Biagioni M Simmons D Dunger DB Nodale M Wilinska ME Amiel SA Hovorka R Safety and efficacy of 24 h closed-loop insulin delivery in well-controlled pregnant women with type 1 diabetes: a randomized crossover case series Diabetes Care 2011 in press 10.2337/dc11-1430 PMC3220861 22011408

[B47] Castle JR Engle JM El YJ Massoud RG Yuen KC Kagan R Ward WK Novel use of glucagon in a closed-loop system for prevention of hypoglycemia in type 1 diabetes Diabetes Care 2010 33 1282 1287 10.2337/dc09-2254 20332355 PMC2875438

[B48] El-Khatib FH Russell SJ Nathan DM Sutherlin RG Damiano ER A bihormonal closed-loop artificial pancreas for type 1 diabetes Sci Transl Med 2010 2 27ra27 10.1126/scitranslmed.3000619 20393188 PMC4242106

[B49] Wilinska ME Chassin LJ Acerini CL Allen JM Dunger DB Hovorka R Simulation environment to evaluate closed-loop insulin delivery systems in type 1 diabetes J Diabetes Sci Technol 2010 4 132 144 20167177 10.1177/193229681000400117 PMC2825634

[B50] Kovatchev BP Breton M Man CD Cobelli C In silico preclinical trials: a proof of concept in closed-loop control of type 1 diabetes J Diabetes Sci Technol 2009 3 44 55 19444330 10.1177/193229680900300106 PMC2681269

[B51] Wilinska ME Budiman ES Taub MB Elleri D Allen JM Acerini CL Dunger DB Hovorka R Overnight closed-loop insulin delivery with model predictive control: assessment of hypoglycemia and hyperglycemia risk using simulation studies J Diabetes Sci Technol 2009 3 1109 1120 20144424 10.1177/193229680900300514 PMC2769888

[B52] Kanderian SS Weinzimer S Voskanyan G Steil GM Identification of intraday metabolic profiles during closed-loop glucose control in individuals with type 1 diabetes J Diabetes Sci Technol 2009 3 1047 1057 20144418 10.1177/193229680900300508 PMC2769900

[B53] Juvenile Diabetes Research Foundation Continuous Glucose Monitoring Study Group Satisfaction with continuous glucose monitoring in adults and youths with Type 1 diabetes Diabet Med 2011 28 9 1118 1122 10.1111/j.1464-5491.2011.03368.x 21692844

[B54] Elleri D Harris J Kumareswaran K Allen JM Haidar A Nodale M Swamy A Wilinska ME Weston J Acerini CL Jackson N Umpleby AM Evans ML Dunger DB Hovorka R Glucose appearance of large slowly-absorbed evening meal containing complex carbohydrates (CHO) in type 1 diabetes (T1D) Diabetologia 2010 53 Suppl 1 S 272

[B55] Weinzimer SA Sherr JL Cengiz E Kim G Carria L Tamborlane WV Effect of adjuvant injected pramlintide on closed-loop automated insulin delivery Diabetes 2011 60 Supll 1 A 253

[B56] Hompesch M Muchmore DB Morrow L Vaughn DE Accelerated insulin pharmacokinetics and improved postprandial glycemic control in patients with type 1 diabetes after coadministration of prandial insulins with hyaluronidase Diabetes Care 2011 34 666 668 10.2337/dc10-1892 21273493 PMC3041204

[B57] Steiner S Hompesch M Pohl R Simms P Flacke F Mohr T Pfutzner A Heinemann L A novel insulin formulation with a more rapid onset of action Diabetologia 2008 51 1602 1606 10.1007/s00125-008-1095-8 18641968 PMC2516197

[B58] Renard E Place J Cantwell M Chevassus H Palerm CC Closed-loop insulin delivery using a subcutaneous glucose sensor and intraperitoneal insulin delivery: feasibility study testing a new model for the artificial pancreas Diabetes Care 2010 33 121 127 10.2337/dc09-1080 19846796 PMC2797956

[B59] Liebl A Hoogma R Renard E Geelhoed-Duijvestijn PH Klein E Diglas J Kessler L Melki V Diem P Brun JM Schaepelynck-Belicar P Frei T A reduction in severe hypoglycaemia in type 1 diabetes in a randomized crossover study of continuous intraperitoneal compared with subcutaneous insulin infusion Diabetes Obes Metab 2009 11 1001 1008 10.1111/j.1463-1326.2009.01059.x 19740082

[B60] Kumareswaran K Elleri D Allen JM Harris J Xing D Kollman C Nodale M Murphy HR Amiel SA Heller SR Wilinska ME Acerini CL Evans ML Dunger DB Hovorka R Meta-analysis of overnight closed-loop randomised studies in children and adults with type 1 diabetes: the Cambridge cohort J Diabetes Sci Technol 2011 in press 10.1177/193229681100500606 PMC3262701 22226252

[B61] Choudhary P Shin J Wang Y Evans ML Hammond PJ Kerr D Shaw JA Pickup JC Amiel SA Insulin pump therapy with automated insulin suspension in response to hypoglycemia: reduction in nocturnal hypoglycemia in those at greatest risk Diabetes Care 2011 34 2023 2025 10.2337/dc10-2411 21868778 PMC3161284

[B62] Danne T Kordonouri O Holder M Haberland H Golembowski S Remus K Blasig S Wadien T Zierow S Hartmann R Thomas A Prevention of hypoglycemia by using low glucose suspend function in sensor-augmented pump therapy Diabetes Technol Ther 2011 13 1 6 10.1089/dia.2010.0116 21827318

[B63] Kovatchev B Cobelli C Renard E Anderson S Breton M Patek S Clarke W Bruttomesso D Maran A Costa S Avogaro A Dalla MC Facchinetti A Magni L De NG Place J Farret A Multinational study of subcutaneous model-predictive closed-loop control in type 1 diabetes mellitus: summary of the results J Diabetes Sci Technol 2010 4 1374 1381 21129332 10.1177/193229681000400611 PMC3005047

[B64] Steil GM Rebrin K Darwin C Hariri F Saad MF Feasibility of automating insulin delivery for the treatment of type 1 diabetes Diabetes 2006 55 3344 3350 10.2337/db06-0419 17130478

